# Glycation of Salivary Aldehyde Dehydrogenase: Emerging Molecular Mechanisms and Clinical Implications in Oral Disease

**DOI:** 10.3390/life16030463

**Published:** 2026-03-12

**Authors:** Masood Alam Khan, Hina Younus

**Affiliations:** 1 Department of Basic Health Sciences, College of Applied Medical Sciences, Qassim University, Buraydah 51412, Saudi Arabia; 2 Interdisciplinary Biotechnology Unit, Faculty of Life Sciences, Aligarh Muslim University, Aligarh 202002, India

**Keywords:** salivary ALDH, oxidative stress, methylglyoxal, carbonyl stress, oral biomarkers, oral health

## Abstract

Salivary aldehyde dehydrogenases (ALDHs), particularly ALDH3A1 and ALDH1A1, serve as frontline enzymatic defenses in the oral cavity, detoxifying reactive aldehydes generated through metabolic activity, microbial fermentation, and environmental exposures. These enzymes are essential for maintaining redox homeostasis, mucosal integrity, and immune modulation. However, under chronic metabolic stress, such as in diabetes, oral inflammation, and cancer, salivary ALDHs become vulnerable to non-enzymatic glycation by reactive carbonyl species like methylglyoxal. This modification impairs cofactor binding, catalytic activity, and structural stability, thereby compromising detoxification capacity at a time of heightened aldehyde burden. This review provides the first insights into ALDH glycation and particularly that of salivary ALDH, examining its structural mechanisms, disease-specific consequences, and emerging protective strategies. Special focus is given to natural compounds, including curcumin, thymoquinone, resveratrol, carnosine, and EGCG, that prevent glycation or restore ALDH function via carbonyl scavenging, antioxidant activation, and NAD^+^/SIRT1 pathway modulation. We also highlight critical research gaps, such as the absence of site-specific glycation maps, lack of salivary gland-based models, and limited availability of ALDH3A1-specific activators. Importantly, we propose that the glycation status of salivary ALDHs may serve as a non-invasive biomarker of oxidative stress and therapeutic response in metabolic and inflammatory disorders. By bridging biochemical insights with translational potential, this review establishes ALDH glycation as a mechanistic and clinically actionable axis in oral and systemic health.

## 1. Introduction

Saliva is increasingly recognized not only for its roles in lubrication, digestion, and antimicrobial defense but also as a dynamic, non-invasive diagnostic medium that reflects both oral and systemic health states [1]. It contains a complex array of biomolecules, enzymes, metabolites, extracellular vesicles, and exfoliated cells, that offer insight into redox status, tissue integrity, and metabolic stress [2]. Among these, salivary aldehyde dehydrogenases (ALDHs) play a pivotal role in maintaining epithelial homeostasis and oxidative balance by detoxifying reactive aldehydes generated from lipid peroxidation, microbial fermentation, or environmental sources [3,4]. These NAD(P)^+^-dependent enzymes, including isoforms such as ALDH3A1 and ALDH1A1, are expressed in oral epithelia and salivary glands [5,6]. Both ALDH1A1 and ALDH3A1 are expressed in oral tissues, but they differ in functional emphasis. ALDH3A1 is the predominant isoform in salivary gland epithelia and primarily detoxifies lipid peroxidation-derived aldehydes such as 4-HNE, thereby serving as a frontline defense against oxidative stress. In contrast, ALDH1A1 participates more broadly in intracellular aldehyde metabolism and retinoic acid biosynthesis, linking it to cellular differentiation and signaling pathways [6,7]. Given that much of the available glycation evidence originates from systemic ALDH2 or ALDH1A1 studies, extrapolation to salivary ALDH3A1 should be undertaken cautiously, highlighting the need for isoform-specific investigation in oral tissues. ALDH3A1 is abundantly expressed in epithelial tissues and plays a well-established cytoprotective role against oxidative and aldehyde-induced stress [7,8,9]. Experimental studies in human corneal epithelial cells demonstrate that stable ALDH3A1 expression attenuates apoptosis, reduces caspase-3 activation and PARP cleavage, limits 4-HNE–protein adduct formation, and enhances NAD(P)H production during aldehyde exposure, underscoring its central role in epithelial redox homeostasis [10,11]. Extending these findings to oral pathology, clinical evidence shows that ALDH3A1 expression is significantly reduced in oral squamous cell carcinoma (OSCC), where lower levels correlate with poor differentiation, nodal involvement, and advanced disease stage [12]. Moreover, restoration of ALDH3A1 suppresses proliferation, migration, invasion, and epithelial-to-mesenchymal transition in OSCC cells, partly through modulation of the IL-6/STAT3 signaling pathway, supporting its tumor-suppressive and detoxifying functions in oral epithelium [13]. Despite this expanding epithelial and oncologic evidence, the specific regulation and glycation susceptibility of ALDH isoforms in saliva remain insufficiently characterized compared with their systemic counterparts.

ALDHs are structurally sensitive enzymes that require an intact catalytic cysteine and NAD(P)^+^-binding domain to function [14]. Glycation near these domains could impede cofactor binding or disrupt enzymatic turnover. This possibility is especially relevant in the oral cavity, where salivary enzymes are exposed to fluctuating glucose levels, aldehyde stress, and microbial metabolites. Moreover, in chronic conditions such as diabetes, ALDH activity in saliva appears to follow a biphasic pattern, with initial upregulation followed by a progressive decline in parallel with increased oxidative and carbonyl stress. Notably, this observation originates from a cross-sectional clinical study involving 161 patients with diabetes, which currently represents the primary direct clinical evidence supporting alterations in salivary ALDH activity in this population [15]. It is important to emphasize that this proposed biphasic pattern is therefore based largely on a single observational study and requires independent validation in larger and longitudinal cohorts before being considered a consistently established biological phenomenon. There is increasing concern over how post-translational modifications, especially non-enzymatic glycation, affect ALDH activity. Triggered by reactive carbonyl species such as methylglyoxal under hyperglycemic or inflammatory conditions, glycation can alter critical lysine, arginine, or cysteine residues, resulting in structural distortion, disrupted cofactor binding, and diminished enzymatic efficiency [16]. Such modifications have been well studied in proteins like hemoglobin and albumin [17,18], but their effects on salivary ALDHs remain largely unexplored. The oral cavity presents a unique biochemical environment where ALDHs are continuously exposed to fluctuating glucose, oxidative byproducts, and microbial aldehydes. The decline of salivary ALDH may lead to aldehyde accumulation, epithelial damage, redox imbalance, and inflammatory signaling, all of which accelerate disease progression.

Notably, natural compounds such as curcumin, resveratrol, carnosine, and thymoquinone have shown promise in mitigating glycation or restoring ALDH activity by scavenging carbonyl species, activating Nrf2 and SIRT1 pathways, and stabilizing enzyme structure; however, most of these effects have been demonstrated in vitro or in systemic models, and their direct efficacy in preserving salivary ALDH remains to be established [19,20,21,22]. These agents offer a translational avenue for developing targeted salivary therapeutics, via oral rinses, lozenges, or gels, aimed at preserving ALDH function and redox integrity. In this review, we explore the emerging landscape of ALDH glycation and particularly that of salivary ALDH, from molecular mechanisms to clinical implications. We highlight natural compounds as protective agents, identify key research gaps such as isoform-specific glycation mapping and diagnostic development, and propose salivary ALDH glycation status as a novel biomarker of oral–systemic disease. This framework sets the stage for therapeutic innovation at the intersection of redox biology, salivary diagnostics, and metabolic health. This article is a narrative review. A structured literature search was conducted using PubMed, Web of Science, and Embase databases covering the period from 2000 to 2025. Keywords included combinations of “salivary ALDH,” “ALDH3A1,” “glycation,” “methylglyoxal,” “advanced glycation end products,” “oral disease,” “oxidative stress,” and “natural compounds.” Priority was given to peer-reviewed original research articles, clinical studies involving salivary samples, mechanistic in vitro investigations, and relevant animal models. Studies focusing exclusively on unrelated ALDH isoforms without mechanistic relevance to glycation were excluded. Given the exploratory and integrative objective of this review, a PRISMA framework was not applied.

## 2. Molecular Mechanisms of ALDH Glycation

Aldehyde dehydrogenases (ALDHs) are structurally conserved enzymes that catalyze the NAD(P)^+^-dependent oxidation of aldehydes into their less reactive carboxylic acid derivatives. Central to this function is the integrity of their active-site cysteine, the Rossmann fold cofactor-binding domain, and the oligomerization interface that ensures structural stability and enzymatic efficiency [14]. Disruption of these features, by mutation, oxidative stress, or non-enzymatic glycation, can compromise catalytic activity [23]. Glycation, an irreversible post-translational modification driven by elevated glucose or carbonyl species such as methylglyoxal, is particularly deleterious in this context as it targets critical lysine and arginine residues, leading to conformational disruption, loss of function, and accelerated degradation.

### 2.1. Structural and Chemical Basis of ALDH Glycation

**Figure 1:**
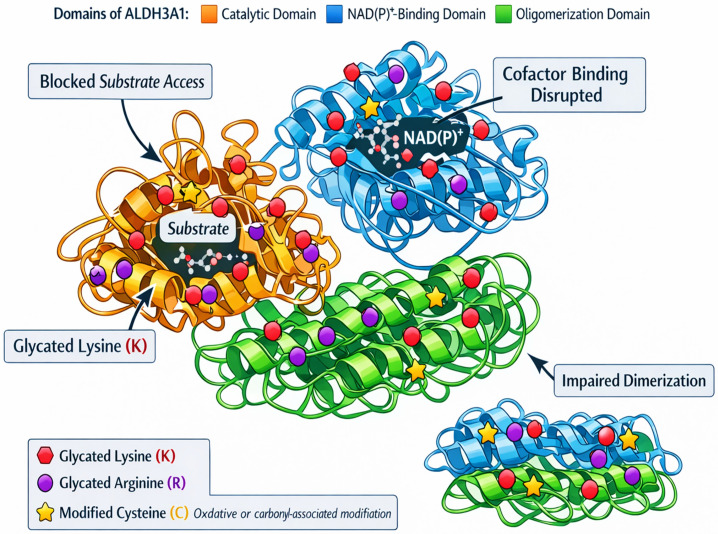
Structural vulnerability of ALDH3A1 to glyco-oxidative stress. The schematic illustrates the domain architecture of ALDH3A1, highlighting the catalytic domain (orange), NAD(P)^+^-binding domain (blue), and oligomerization domain (green). Canonical glycation-prone residues, lysine (K, red) and arginine (R, purple), are indicated as primary targets of non-enzymatic carbonyl modification. The catalytic cysteine (C, yellow) is shown separately to denote its susceptibility to oxidative or carbonyl-associated modification under glyco-oxidative stress rather than classical Maillard glycation. Modification of lysine and arginine residues may impair substrate access, disrupt NAD(P)^+^ binding, and destabilize dimerization interfaces, compromising ALDH3A1 detoxification capacity.

**Figure 2:**
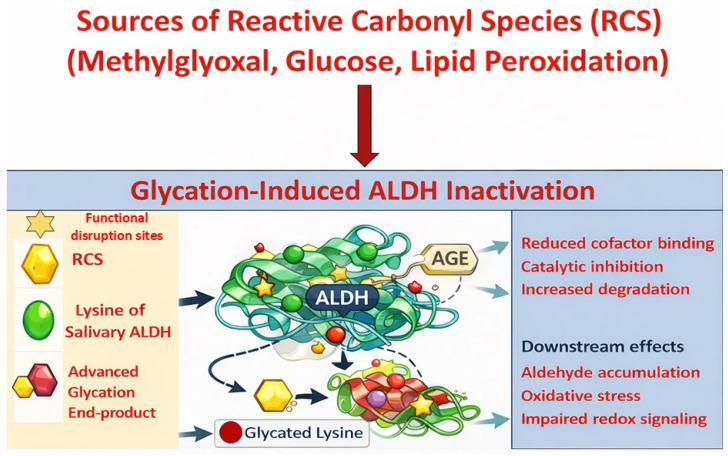
Mechanism of glycation-induced ALDH inactivation and its consequences. Reactive carbonyl species (RCS) generated from glucose, methylglyoxal, and lipid peroxidation during oxidative stress drive the non-enzymatic glycation of lysine residues on ALDH enzymes. This leads to advanced glycation end-product (AGE) formation, resulting in impaired ALDH function through reduced cofactor binding, catalytic inhibition, and increased degradation. ALDH inactivation contributes to aldehyde accumulation, oxidative stress, and disrupted redox signaling, key factors in the progression of metabolic and inflammatory diseases.

ALDH isoforms expressed in saliva, notably ALDH3A1 and ALDH1A1, exhibit structural motifs that render them susceptible to glycation under hyperglycemic or oxidative stress conditions [14,15]. These enzymes share a conserved architecture composed of three major domains: a catalytic domain, a cofactor-binding Rossmann fold, and an oligomerization domain [24]. Lysine and arginine residues are prominently localized near the cofactor cleft and domain interfaces, making them prime glycation sites. Glycation proceeds via nucleophilic attack on these residues by reactive dicarbonyls like methylglyoxal, forming Schiff base intermediates, which evolve through Amadori rearrangement into stable AGEs (Figure 1). These modifications hinder NAD(P)^+^ binding, destabilize protein structure, and disrupt substrate access. Although direct glycation site mapping on salivary ALDH isoforms has not yet been performed, structural homology with other ALDH family members suggests comparable vulnerabilities within catalytic and cofactor-binding domains; however, this inference is based on structural and biochemical studies of non-salivary isoforms such as ALDH2 and ALDH1A1 and therefore remains to be experimentally validated in salivary ALDHs (Figure 2). In the oral cavity, characterized by dynamic glucose flux, aldehyde generation, and oxidative insults, these structural susceptibilities may impair detoxification efficiency during metabolic stress.

### 2.2. Glycation-Induced Functional Consequences

Glycation of ALDHs in saliva leads to a loss of detoxifying and redox-buffering functions. Covalent modification of active-site residues alters substrate access and catalytic dynamics, reducing the breakdown of harmful aldehydes such as 4-HNE, MDA, and acetaldehyde [25,26]. This results in oxidative stress, DNA damage, and inflammatory signaling. Furthermore, ALDH-mediated NAD(P)H regeneration supports redox systems like glutathione and thioredoxin. Glycation-induced ALDH inactivation impairs these processes, disrupting Nrf2 activation while promoting NF-κB-driven inflammation. In epithelial tissues, ALDH3A1 also contributes to progenitor cell renewal. Its glycation may thus reduce epithelial integrity and repair, particularly in diseases like diabetes where ALDH shows a biphasic activity pattern, with early upregulation followed by decline due to cumulative glycation [15,27]. However, the evidence supporting this dynamic shift remains limited, as it is derived from a single clinical study. Additional mechanistic and longitudinal investigations are necessary to confirm whether this biphasic pattern represents a consistent biological phenomenon in diabetic populations.

### 2.3. Experimental and Clinical Evidence for ALDH Glycation

Clinical studies in diabetic patients demonstrate reduced salivary ALDH activity accompanied by increased AGEs and oxidative DNA damage markers such as 8-OHdG; however, this evidence is currently limited to a small number of salivary-based clinical investigations and warrants validation in larger and longitudinal cohorts [15]. In vitro investigations further show that ALDH1 and ALDH2 exhibit diminished activity in cardiomyocytes following methylglyoxal exposure or high-glucose treatment; nevertheless, these findings arise from systemic cardiac models and cannot be directly assumed to reflect salivary ALDH behavior [28,29]. Furthermore, simultaneous deletion of ALDH3A1 and GLO1 in animal models exacerbates dicarbonyl stress and proteasomal dysfunction; however, these observations are derived from non-salivary tissues and therefore provide mechanistic support rather than direct evidence for salivary ALDH glycation [30]. These findings highlight the likely susceptibility of ALDH3A1 to glycation in oral settings and support further investigation.

### 2.4. Microenvironmental Influences in the Oral Cavity

The oral cavity amplifies glycation risk due to the presence of systemic glucose, microbial fermentation products, and environmental aldehydes. Fermentative microbiota can generate methylglyoxal and acetaldehyde, while smoking and alcohol introduce exogenous aldehydes, increasing glycation pressure [31,32]. Compounding this, hyposalivation, as in aging or xerostomia, reduces dilution of these agents, further concentrating glycating species [33]. Unlike systemic ALDHs, salivary ALDHs operate extracellularly or within exosomes, exposing them to highly variable biochemical environments. These contextual differences underscore the need to explore vesicle-mediated protection, glycation-resistant isoforms, or delivery of enzyme stabilizers in oral formulations.

## 3. Disease Associations of Glycated Salivary ALDH

**Figure 3:**
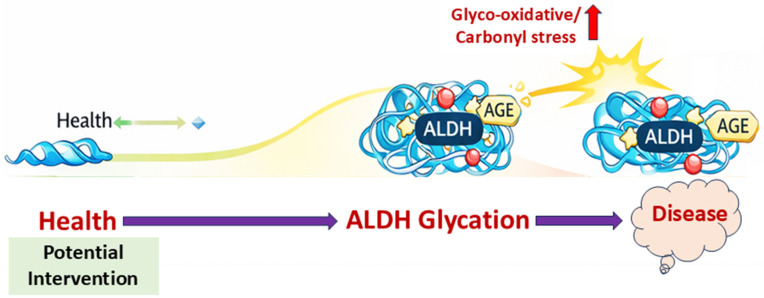
Disease progression model linking ALDH glycation to oral and systemic diseases. This schematic figure illustrates the transition from normal salivary ALDH function, maintaining aldehyde detoxification and redox balance, to progressive disease states under chronic glyco-oxidative stress. Sustained hyperglycemia and carbonyl burden promote ALDH glycation, leading to impaired enzymatic activity, aldehyde accumulation, and inflammatory signaling. Certain stages within the timeline represent mechanistically inferred or hypothesis-driven transitions.

The enzymatic function of salivary ALDHs, particularly ALDH3A1 and ALDH1A1, is vital for maintaining mucosal detoxification, redox equilibrium, and epithelial integrity in the oral environment (Figure 3). Under chronic metabolic stress, common in diabetes, cancer, and periodontal diseases, these enzymes are increasingly vulnerable to post-translational glycation, which disrupts their catalytic roles. This section examines the disease-specific consequences of ALDH glycation, emphasizing its pathophysiological implications and translational relevance.

### 3.1. Diabetes and Oral Complications

In the early stages of diabetes, available clinical evidence indicates that salivary ALDHs are upregulated in response to rising levels of glucose-derived aldehydes and oxidative stress, reflecting a compensatory protective mechanism; however, this conclusion is based on limited salivary-specific studies and requires confirmation in broader clinical populations [15]. However, this compensatory upregulation is transient. Persistent hyperglycemia promotes the glycation of lysine residues near the NAD(P)^+^-binding site of ALDHs, impairing enzymatic activity [34]. This reduces detoxification of cytotoxic aldehydes like 4-HNE and methylglyoxal, leading to the accumulation of AGEs and oxidative stress biomarkers, including 8-OHdG and malondialdehyde (Figure 3). These biochemical disruptions correlate with impaired wound healing, periodontal inflammation, and xerostomia, hallmarks of oral diabetic complications. Under high-glucose conditions, cardiac fibroblasts exhibit reduced ALDH2 expression and increased proliferation, whereas activation of ALDH2 with the agonist Alda-1 restores ALDH2 levels and significantly suppresses fibroblast proliferation, highlighting a protective, anti-fibrotic role of ALDH2 in hyperglycemic stress [35]. Glycation may also impair NAD(P)H regeneration, compromise antioxidant systems and disrupt Nrf2 signaling, while favoring pro-inflammatory cascades via NF-κB. The biphasic pattern of ALDH activity, initial elevation followed by glycation-induced decline, may serve as an early indicator of glycemic burden and mucosal vulnerability.

### 3.2. Periodontal Inflammation and Precancerous Lesions

Chronic periodontitis and precancerous lesions like leukoplakia and OLP involve a sustained inflammatory milieu rich in ROS, aldehydes, and immune mediators [36]. ALDH3A1 is often overexpressed in these tissues, potentially reflecting its dual role in detoxification and epithelial stemness. Prolonged carbonyl stress can lead to ALDH glycation, compromising enzymatic function despite elevated expression [37]. This uncoupling of ALDH activity from protein levels may promote the survival of stem-like epithelial cells with impaired detoxification capacity, increasing their susceptibility to dysplastic transformation. Glycation may interfere with redox-sensitive signaling (e.g., p53, Notch), weaken apoptosis checkpoints, and enhance proliferative signaling, thus facilitating epithelial plasticity and progression toward dysplasia [38]. Persistent ROS from dysfunctional ALDH may activate NF-κB and STAT3, fostering a microenvironment conducive to extracellular matrix degradation and tumor initiation.

### 3.3. Cancer and Treatment-Induced Damage

In oral squamous cell carcinoma, ALDH-positive cancer stem-like cells contribute to therapy resistance and recurrence; however, this evidence is derived primarily from tumor tissue studies and does not specifically address salivary gland-derived ALDH isoforms [39,40]. ALDH activity supports DNA repair, redox buffering, and aldehyde detoxification, sustaining CSC viability under stress. However, cancer therapies like radiation and chemotherapy elevate local levels of methylglyoxal and glucose, which can promote ALDH glycation [41]. This post-translational modification can impair residual ALDH function in CSCs, weakening detoxification capacity and increasing susceptibility to oxidative damage. It is important to emphasize that the potential therapeutic implications of ALDH glycation in oral cancer remain speculative. Although selective glycation of ALDH isoforms could theoretically impair cancer stem cell (CSC) resilience by altering enzymatic detoxification capacity, this hypothesis has not yet been experimentally validated in salivary or oral cancer models. Similarly, the possibility that glycated ALDH isoforms may generate neoantigenic epitopes capable of modulating tumor immunogenicity represents a conceptual framework rather than an established mechanism. Further mechanistic and translational studies are required to determine whether such modifications meaningfully influence oral cancer progression or therapeutic response [42,43]. Non-invasive monitoring of salivary ALDH glycation through activity assays and glycation-specific markers may help assess mucosal recovery, treatment efficacy, and recurrence risk. This positions glycated ALDHs as both biomarkers and therapeutic targets in oral oncology.

## 4. Protective Role of Natural Compounds in Preserving ALDH Function Under Glycation Stress

Maintaining salivary ALDH function under chronic glyco-oxidative stress is a growing concern in oral and systemic redox health. Salivary isoforms like ALDH3A1 and ALDH1A1 play vital roles in neutralizing toxic aldehydes, yet under diabetic, inflammatory, or aging conditions, elevated levels of reactive carbonyl species (RCS), especially methylglyoxal (MG), initiate glycation at nucleophilic sites near the catalytic or cofactor-binding domains. This leads to enzyme misfolding, activity loss, and proteasomal degradation [14,15]. Thus, preserving ALDH integrity requires coordinated strategies targeting glycation prevention, structural protection, and enzymatic restoration.

### 4.1. Mechanistic Rationale for Natural Compound Use

**Figure 4:**
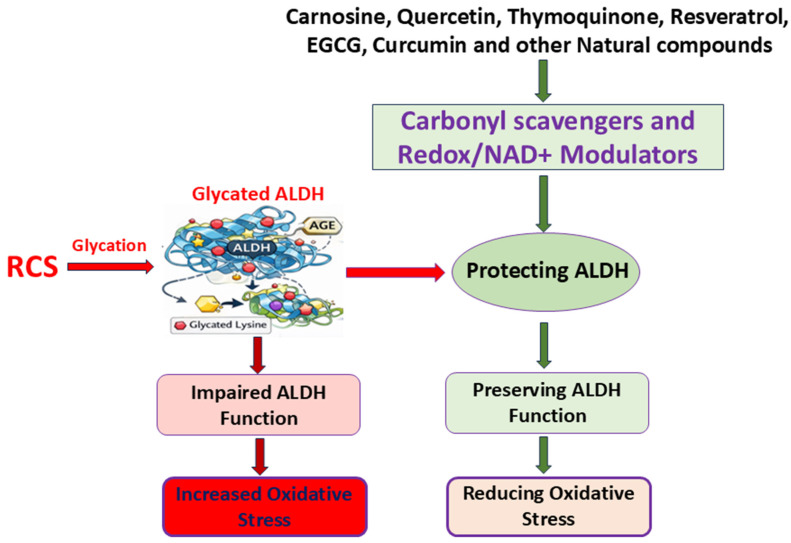
Protective mechanisms of natural compounds against ALDH glycation. Natural compounds counteract glycation-induced ALDH inactivation through multiple pathways: carbonyl scavengers (e.g., carnosine, curcumin) neutralize reactive carbonyl species; redox/NAD^+^ modulators (e.g., resveratrol, quercetin) support cofactor balance and antioxidant defenses; and dual-function polyphenols (e.g., EGCG, thymoquinone) activate SIRT1 and Nrf2 pathways. These actions preserve ALDH function, reduce oxidative stress, and prevent downstream effects such as aldehyde accumulation and impaired redox signaling.

Natural compounds, including polyphenols, quinones, and dipeptides, offer protective benefits that extend beyond general antioxidant action (Figure 4). These include carbonyl scavenging, metal ion chelation, ROS neutralization, and modulation of redox pathways. Their compatibility with oral formulations (e.g., rinses, lozenges, mucoadhesive gels) makes them especially suitable for salivary-targeted applications.

#### 4.1.1. Early Glycation Interception via Carbonyl Trapping

Several bioactives directly trap MG and glyoxal, the key precursors in AGE formation, thereby interrupting glycation at its earliest stage. Carnosine, a naturally occurring dipeptide abundant in salivary glands, neutralizes dicarbonyls via Michael addition, preventing covalent modification of lysine residues critical for ALDH activity. It reduces pentosidine and malondialdehyde (MDA) formation and preserves protein integrity [44]. In vivo studies demonstrate that carnosine attenuates glyco-oxidative damage in serum, liver, and brain tissues of D-galactose-treated rats by lowering ROS and lipid peroxidation; however, these findings are derived from systemic animal models rather than salivary tissues, and their relevance to salivary ALDH protection remains inferential [45]. Curcumin, the principal curcuminoid from *Curcuma longa*, forms 1:1 adducts with MG, chelates Fe^2+^/Cu^2+^, and suppresses Amadori rearrangements. In endothelial cell models, curcumin reduces AGE formation, downregulates inflammatory mediators, and improves viability under methylglyoxal stress; however, these findings are derived from in vitro endothelial systems and have not yet been directly validated in salivary epithelial or glandular models [46]. Thymoquinone binds near Cys243 of ALDH3A1 and enhances substrate affinity and catalytic turnover without altering secondary structure; however, these findings are based on in vitro recombinant salivary ALDH3A1 studies and require confirmation in native salivary gland or clinical contexts [19]. It also preserves SOD activity under glycation conditions, minimizing AGE accumulation and fibril formation [47,48].

#### 4.1.2. Enzyme Stabilization and NAD(P)^+^ Preservation

Beyond intercepting glycation, certain natural compounds stabilize ALDH conformation and help maintain cofactor homeostasis, both essential for sustained detoxification. Resveratrol, a grape-derived stilbene, activates SIRT1 and upregulates NAMPT, boosting NAD^+^ pools vital for ALDH catalysis [4,49]. Resveratrol has been shown to stabilize ALDH2 and improve enzymatic efficiency under stress conditions; however, this evidence is derived from systemic ALDH2 models, and its applicability to salivary ALDH3A1 is inferred based on conserved NAD^+^-binding architecture rather than direct experimental validation [50]. In methylglyoxal-treated rats, resveratrol reduces protein carbonyls, AOPP, TBARS, and AGEs in plasma and liver tissues; however, these findings are derived from systemic in vivo models and do not directly demonstrate protective effects in salivary tissues or on salivary ALDH isoforms [51]. Epigallocatechin gallate (EGCG), the key catechin in green tea, exhibits multi-targeted anti-glycation effects: it traps MG, chelates redox-active metals, scavenges ROS, and reverses glycation-induced fluorescence under high glucose [52,53]. It also protects neuronal cells from MG-induced apoptosis by regulating MAPK pathways [54] and activates Nrf2, enhancing endogenous antioxidant defense and suppressing RAGE under diabetic stress [55]. Quercetin, a dietary flavonol, inhibits AGE formation through combined MG scavenging, metal ion chelation, and ROS neutralization [56]. By reducing 4-HNE and MDA, potent ALDH inhibitors, quercetin supports redox homeostasis and detoxification [57]. Its binding to albumin also prevents glycation-induced structural damage [58].

#### 4.1.3. Mitochondrial and Redox Network Support

ALDH protection also relies on systemic support. Glycation stress often coincides with mitochondrial dysfunction, excessive ROS, and diminished antioxidant capacity. Resveratrol promotes mitochondrial biogenesis and restores the NAD^+^/NADH balance, reducing aldehyde accumulation [22]. EGCG stabilizes mitochondrial membrane potential and triggers Nrf2-mediated gene expression, enhancing antioxidant defenses [52]. Quercetin prevents lipid peroxidation and ferroptosis through the SIRT1/Nrf2/GPx4 axis, contributing to long-term ALDH preservation [57]. Together, these agents support a multi-pronged defense that sustains ALDH function under carbonyl stress.

### 4.2. Therapeutic Integration and Novel Delivery Strategies

The bioavailability of natural compounds, including polyphenols in the oral cavity, is limited due to too-short residence time in the mouth before being swallowed, and for some there is an absence of a local uptake mechanism. In vitro studies generally use µM to mM concentrations of natural compounds; however, the clinical concentrations achieved in vivo are often significantly lower, necessitating higher doses or specialized delivery systems to be effective. To realize clinical translation, natural compounds must be optimized for local oral delivery. Formulations such as mucoadhesive gels, oral rinses, lozenges, or nanoparticle-encapsulated phytochemicals can achieve high tissue concentrations with minimal systemic effects, ideal for ALDH3A1, which localizes to salivary gland epithelia [7,27]. Combination approaches may enhance efficacy. For instance, pairing carnosine with EGCG could unite carbonyl scavenging with long-term antioxidant effects. Similarly, curcumin–resveratrol combinations have shown synergistic SIRT1 activation and AGE suppression in preclinical models [22]. These strategies hold promise not only for preventive care but also as adjunctive treatments in conditions like diabetes, chronic periodontitis, or post-radiation mucositis, where ALDH activity is compromised. Although many naturally occurring phytochemicals demonstrate favorable safety profiles at dietary levels, dose-dependent toxicities have been reported for certain compounds. For example, high-dose curcumin has been associated with hepatotoxicity in rare cases, concentrated EGCG supplements have raised concerns regarding liver injury, and thymoquinone exhibits a relatively narrow therapeutic window [59,60,61]. Therefore, careful dose optimization and safety evaluation are essential before clinical translation.

### 4.3. Outlook and Unmet Needs

Despite encouraging mechanistic and preclinical data, important limitations remain. Only a limited number of studies have directly characterized glycation-specific modifications on salivary ALDH isoforms, and protective effects of natural compounds have rarely been validated in salivary gland-relevant models. Rigorous confirmation in tissue-appropriate systems is needed, including mass spectrometry-based glycopeptide mapping to identify modification hotspots, development of salivary organoid or 3D epithelial platforms to replicate the oral microenvironment, and implementation of ALDH3A1-specific activity assays to distinguish isoform-selective effects. In parallel, the discovery of selective ALDH3A1 activators, potentially inspired by phytochemical scaffolds, remains an underdeveloped but promising avenue in enzyme-targeted drug design.

Importantly, while natural compounds demonstrate multi-targeted mechanisms capable of intercepting carbonyl stress, stabilizing enzyme structure, and modulating redox signaling, their clinical translation remains premature. To date, no clinical trials have specifically evaluated natural compounds for preserving or restoring salivary ALDH activity in oral tissues, highlighting a clear translational gap between mechanistic rationale and therapeutic validation. Addressing this gap will require carefully designed human studies, dose-optimization strategies, and safety profiling tailored to oral delivery systems. If successfully translated, salivary-targeted modulation of ALDH activity could redefine the management of redox-driven oral diseases and position ALDH3A1 as a tractable enzymatic target within precision oral medicine.

## 5. Novel Research Gaps and Future Directions

**Figure 5:**
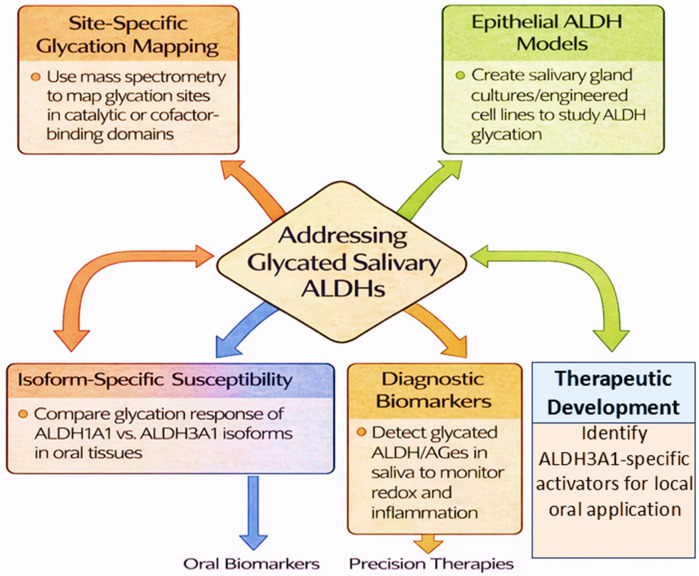
Novel research gaps and future directions in glycated salivary ALDHs. This flowchart summarizes key unmet areas in understanding salivary ALDH glycation, including site-specific glycation mapping, development of physiologically relevant epithelial models, isoform-specific susceptibility of ALDH1A1 versus ALDH3A1, diagnostic biomarker potential in saliva, and the need for ALDH3A1-targeted therapeutic development. Together, these priorities outline a translational roadmap linking structural biology, redox regulation, diagnostics, and precision oral therapeutics.

While the glycation of proteins is increasingly recognized as a pivotal contributor to metabolic and inflammatory disease pathology, its impact on salivary ALDHs remains largely unexplored. Addressing this understudied frontier requires bridging several critical knowledge gaps spanning structural characterization, model development, isoform-specific vulnerability, and translational application (Figure 5). Across the preceding sections, mechanistic insights are derived from a combination of salivary-specific clinical data [9] and systemic or in vitro ALDH isoform studies, underscoring the current reliance on extrapolated evidence and the urgent need for direct glycation mapping in salivary ALDH isoforms.

First, site-specific glycation mapping of salivary ALDHs has yet to be undertaken. Identifying the precise lysine and arginine residues targeted by glycation, especially within catalytic or NAD(P)^+^-binding domains, will clarify mechanistic vulnerabilities and guide rational design of glycation-resistant enzyme variants or small-molecule stabilizers. Mass spectrometry-based proteomic techniques, including glycopeptide profiling and AGE-specific enrichment protocols, should be applied directly to salivary ALDH isoforms rather than inferred from hepatic or recombinant models (Figure 5).

Second, the field lacks physiologically relevant models to study ALDH glycation in the salivary microenvironment. Existing studies rely heavily on systemic isoforms (e.g., ALDH2) in hepatic or cardiovascular contexts. To simulate salivary conditions, characterized by fluctuating glucose, aldehyde load, and microbial metabolites, organotypic salivary gland cultures, 3D epithelial co-cultures, or CRISPR-engineered ALDH3A1-expressing cell lines are urgently needed. Such platforms would allow investigation of glycation kinetics, post-translational regulation, and the efficacy of natural or synthetic protective agents in an epithelial context (Figure 5).

Third, isoform-specific susceptibility to glycation in the salivary compartment remains undefined. While ALDH2 has been extensively studied in cardiovascular and hepatic systems, the comparative sensitivity of ALDH1A1 versus ALDH3A1 to carbonyl stress in oral tissues is unknown. Dissecting these differences could uncover unique roles in maintaining mucosal homeostasis and influencing disease susceptibility, particularly in diabetes, precancerous lesions, and post-radiation xerostomia (Figure 5).

In parallel, the diagnostic potential of glycated salivary ALDHs remains untapped. Since ALDH activity reflects both systemic metabolic control and local oxidative stress, quantifying its glycation-modified forms may provide a non-invasive biomarker for redox imbalance, oral inflammation, or treatment-induced tissue damage. Coupling enzymatic assays with detection of AGE-adducts or mass-spectrometry-defined glycation hotspots could enable point-of-care diagnostic tools for early detection and therapeutic monitoring (Figure 5).

Finally, therapeutic development remains disproportionately focused on ALDH2, with few efforts aimed at modulating ALDH3A1, the dominant isoform in salivary epithelia. There is a pressing need for discovery of isoform-specific ALDH3A1 activators that can stabilize enzyme conformation under glyco-oxidative stress. High-throughput screening for such compounds, ideally optimized for local oral delivery through mucoadhesive systems or rinse formulations, represents a promising translational pathway (Figure 5).

Together, these directions highlight a multidisciplinary research agenda, integrating structural biology, redox enzymology, oral immunology, pharmacology, and clinical diagnostics, to fully elucidate the role of ALDH glycation in oral and systemic health. Addressing these gaps will be pivotal for advancing precision interventions, improving salivary biomarker development, and enhancing therapeutic resilience in high-risk patient populations.

## 6. Conclusions

Glycation of salivary ALDHs, particularly under chronic hyperglycemic and oxidative conditions, represents a critical post-translational modification that impairs the enzyme’s detoxification function, redox balance, and regulatory signaling in the oral cavity. The modification of lysine and arginine residues near catalytic and cofactor-binding domains diminishes ALDH’s ability to clear toxic aldehydes, thereby exacerbating oxidative stress, inflammation, and epithelial dysfunction. These detrimental effects are particularly relevant in diabetes, periodontal disease, precancerous lesions, and oral cancer, where ALDH inactivation may contribute to disease progression and reduced tissue resilience. Importantly, emerging research supports the therapeutic potential of natural compounds such as curcumin, resveratrol, thymoquinone, carnosine, and EGCG, which either prevent glycation, scavenge reactive carbonyl species, or enhance ALDH expression and activity. These agents offer promising strategies for preserving salivary ALDH function and mitigating glycation-associated oral pathologies. Additionally, the glycation status of salivary ALDH may serve as a valuable non-invasive biomarker for monitoring systemic oxidative stress and predicting disease risk or treatment outcomes. Future research should prioritize mass spectrometry-based site mapping, isoform-specific glycation models, and development of glycation-resistant enzyme variants. Collectively, salivary ALDH represents both a therapeutic target and diagnostic tool in the landscape of glycation-driven oral and systemic diseases.

## Data Availability

No new data were created or analyzed in this study.
